# Machine learning enhances the performance of short and long-term mortality prediction model in non-ST-segment elevation myocardial infarction

**DOI:** 10.1038/s41598-021-92362-1

**Published:** 2021-06-18

**Authors:** Woojoo Lee, Joongyub Lee, Seoung-Il Woo, Seong Huan Choi, Jang-Whan Bae, Seungpil Jung, Myung Ho Jeong, Won Kyung Lee

**Affiliations:** 1 Department of Public Health Sciences, Graduate School of Public Health, Seoul National University, Seoul, Republic of Korea; 2 Department of Preventive Medicine, Seoul National University College of Medicine, Seoul, Republic of Korea; 3 Department of Cardiology, School of Medicine, Inha University Hospital, Inha University, Incheon, Republic of Korea; 4 Division of Cardiology, Department of Internal Medicine, Chungbuk National University College of Medicine, Cheongju, Republic of Korea; 5 Chonnam National University Hospital, Gwangju, Republic of Korea; 6 Department of Prevention and Management, School of Medicine, Inha University Hospital, Inha University, 27 Inhang-Ro, Jung-Gu, Incheon, Republic of Korea

**Keywords:** Translational research, Cardiology, Medical research

## Abstract

Machine learning (ML) has been suggested to improve the performance of prediction models. Nevertheless, research on predicting the risk in patients with acute myocardial infarction (AMI) has been limited and showed inconsistency in the performance of ML models versus traditional models (TMs). This study developed ML-based models (logistic regression with regularization, random forest, support vector machine, and extreme gradient boosting) and compared their performance in predicting the short- and long-term mortality of patients with AMI with those of TMs with comparable predictors. The endpoints were the in-hospital mortality of 14,183 participants and the three- and 12-month mortality in patients who survived at discharge. The performance of the ML models in predicting the mortality of patients with an ST-segment elevation myocardial infarction (STEMI) was comparable to the TMs. In contrast, the areas under the curves (AUC) of the ML models for non-STEMI (NSTEMI) in predicting the in-hospital, 3-month, and 12-month mortality were 0.889, 0.849, and 0.860, respectively, which were superior to the TMs, which had corresponding AUCs of 0.873, 0.795, and 0.808. Overall, the performance of the predictive model could be improved, particularly for long-term mortality in NSTEMI, from the ML algorithm rather than using more clinical predictors.

## Introduction

Acute myocardial infarction (AMI) is a leading cause of mortality despite recent advances in percutaneous coronary intervention (PCI) based on the use of drug-eluting stents and pharmacotherapy, including beta-blockers and the renin-angiotensin system blocker^1,2^. A prediction of the severity and prognosis is vital for identifying patients at high risk and providing intensive treatment and monitoring^3^. Traditional risk stratification was based on risk score systems, such as the thrombolysis in myocardial infarction (TIMI), global registry of acute coronary events (GRACE), and acute coronary treatment and intervention outcomes network—Get With The Guidelines (ACTION-GWTG), which extracts the weight from the regression model^3–10^. GRACE and ACTION-GWTG presented a common model for ST-segment elevation myocardial infarction (STEMI) and non-ST-segment elevation myocardial infarction (NSTEMI), whereas TIMI suggested two distinct risk stratifications. Although these models were validated and are commonly accepted tools, concerns have been raised recently because most traditional risk stratifications were developed 20 years ago using randomized controlled trial (RCT) data before the introduction of drug-eluting stents and newer generation antiplatelets^11^. Moreover, the outcomes of the prediction models were limited to short-term mortality, such as in-hospital, 14-day, and 30-day mortality^3,12,13^. Therefore, one review study on conventional risk stratification models suggested that future models would permit more accurate risk stratification^3^.

Recently, machine learning (ML) was suggested to improve the performance of the prediction model because it could overcome the limitations of a regression-based risk score system, including parametric assumption, primary reliance on linearity, and limited capability in examining higher-order interactions^14^. Few attempts have been made to apply ML to risk prediction in patients with AMI, but the attempts made were inconsistent^15,16^. Recent research reported the possibility of performance enhancement using deep learning^11,17^. On the other hand, a direct comparison was not possible because far more predictors were included in the ML models than the traditional methods. Therefore, it is unclear if the performance improvement comes from the machine learning algorithms or the inclusion of more predictors in the ML models. Furthermore, the high computation power and many clinical predictors, which are difficult to extract from the electronic medical records, limit the use of prediction models using deep learning algorithms in clinical practice.

This study compared the performance of ML models in predicting the short- and long-term mortality using comparable predictors in AMI patients with the traditional risk score methods. Furthermore, this study also examined whether adding more predictors to the ML models would improve the performance of the prediction models.

## Results

### Patient enrollment and characteristics

Patients diagnosed with AMI were classified into STEMI and NSTEMI. Of the 5557 patients with STEMI, 273 patients (4.9%) died during the hospital stay (Supplementary Table 1). After excluding those with missing information on the variables during hospital admission, the final dataset for the three- and 12-month mortality contained 4911 survivors at hospital discharge. Among the survivors, 68 and 120 patients died within 3 and 12 months after hospital discharge, giving a mortality rate of 1.4% and 2.4%, respectively. For NSTEMI, 281 patients (3.3%) died after ED arrival among the 8626 patients examined. Of the 7716 survivors, 142 and 306 patients died within three and 12 months after hospital discharge, giving a mortality rate of 1.8% and 4.0%, respectively.

Table 1 lists the demographic characteristics, according to mortality, of the patients before excluding those with missing information during hospital admission. The cumulative 12-month mortality of the study participants was 7.2% and 7.1% in STEMI and NSTEMI, respectively. The differences in the patients’ characteristics according to survival in the STEMI group were similar to those of the NSTEMI group. Patients who survived at the 12-month follow up were younger than those who did not (62.4 vs. 73.9 years for STEMI, 66.3 vs. 76.5 years for NSTEMI). The proportion of female participants in the survival group was lower than those in the death group in both STEMI and NSTEMI. Moreover, those who survived at the 12-month follow up were less likely to have hypertension, diabetes, atrial fibrillation, and a history of MI, PCI, and stroke than those who expired during the 12 months after AMI. On the other hand, they were more likely to have dyslipidemia and be current smokers. Furthermore, those who survived at the 12-month follow up were likely to experience chest pain with sweating, have higher blood pressure at presentation, and lower troponin levels than those who had died by the 12-month follow up. The survival group had a lower proportion of heart failure, cardiogenic shock, left main disease, and three-vessel diseases. The survivors were more likely to take aspirin, beta-blockers, angiotensin-converting enzyme inhibitors, and statin than those who died by the 12-month follow up. In contrast, they were less likely to take oral hypoglycemic agents, warfarin, and non-vitamin K antagonist oral anticoagulants.

**Table 1:** Characteristics of the study population.

	Total	STEMI		NSTEMI	
		Survival	Death	Survival	Death
	N = 14,183	N = 5155	N = 402	N = 8011	N = 615
**Demographic characteristics**					
Age (years)	65.5 ± 12.8	62.4 ± 12.5	73.9 ± 12.3	66.3 ± 12.5	76.5 ± 9.5
Female (%)	3522(24.8%)	895(17.4%)	135(33.6%)	2242(28.0%)	250(40.7%)
Height (m)	165.2 ± 8.8	166.8 ± 8.3	162.9 ± 9.4	164.6 ± 8.9	161.4 ± 9.0
Weight (kg)	66.2 ± 12.6	68.0 ± 12.4	61.5 ± 11.9	65.9 ± 12.5	58.9 ± 11.7
**Medical history**					
Hypertension (%)	7291(51.4%)	2281(44.2%)	223(55.5%)	4353(54.3%)	434(70.6%)
Diabetes mellitus (%)	4286(30.2%)	1205(23.4%)	137(34.1%)	2618(32.7%)	326(53.0%)
Dyslipidemia (%)	1894(13.4%)	589(11.4%)	26(6.5%)	1221(15.2%)	58(9.4%)
Previous MI (%)	1399 (9.9%)	315(6.1%)	34(8.5%)	911 (11.4%)	139(22.6%)
Previous PCI (%)	2052(14.5%)	466(9.0%)	48(11.9%)	1377(17.2%)	161(26.2%)
Stroke (%)	1066(7.5%)	253(4.9%)	44(10.9%)	662 (8.3%)	107(17.4%)
*Smoking*					
Current smoking (%)	5234(36.9%)	2410(46.8%)	101(25.1%)	2614(32.6%)	109(17.7%)
Past smoking (%)	2867(20.2%)	914 (17.7%)	87(21.6%)	1740(21.7%)	126(20.5%)
**Symptom**					
Chest pain (%)	12,474(88.0%)	4893(94.9%)	288(71.6%)	6905(86.2%)	388(63.1%)
Dyspnea (%)	4148(29.2%)	1164(22.6%)	141(35.1%)	2498(31.2%)	345(56.1%)
Loss of awareness (%)	750 (5.3%)	290 (5.6%)	101(25.1%)	302 (3.8%)	57 (9.3%)
Sweat (%)	3841(27.1%)	1742(33.8%)	77(19.2%)	1934(24.1%)	88(14.3%)
Vertigo and systemic weakness (%)	1429(10.1%)	495(9.6%)	47 (11.7%)	797 (9.9%)	90(14.6%)
Epigastric pain (%)	524(3.7%)	183(3.5%)	24 (6.0%)	281 (3.5%)	36 (5.9%)
Radiating pain (%)	3584(25.3%)	1432(27.8%)	69 (17.2%)	2013(25.1%)	70(11.4%)
**Initial presentation**					
Systolic blood pressure (mmHg)	132.5 ± 31.2	128.1 ± 30.8	100.9 ± 46.7	137.9 ± 28.3	119.5 ± 36.1
Diastolic blood pressure (mmHg)	79.0 ± 19.3	77.6 ± 19.7	61.3 ± 30.0	81.5 ± 17.2	70.0 ± 22.2
Heart rate (bpm)	79.8 ± 20.7	76.5 ± 20.4	77.7 ± 35.1	81.2 ± 19.1	89.0 ± 25.5
**Laboratory findings**					
Troponin I (ng/mL)	10.5 ± 34.7	14.5 ± 42.8	28.7 ± 56.5	7.1 ± 26.1	12.3 ± 38.9
Troponin T (ng/mL)	7.3 ± 70.1	6.0 ± 74.4	30.9 ± 182.1	6.7 ± 51.7	11.5 ± 85.7
Creatinine (ng/dL)	1.3 ± 1.8	1.1 ± 1.2	1.6 ± 1.4	1.3 ± 1.8	2.3 ± 4.1
Hemoglobin (g/dL)	13.7 ± 2.2	14.3 ± 1.9	12.3 ± 2.3	13.5 ± 2.2	11.4 ± 2.4
**Clinical manifestation**					
Heart failure (%)	1267 (8.9%)	265 (5.1%)	89(22.1%)	708 (8.8%)	205(33.3%)
Cardiogenic shock (%)	809 (5.7%)	364 (7.1%)	161(40.0%)	190 (2.4%)	94(15.3%)
**Echocardiographic finding**					
LV ejection fraction	51.6 ± 11.6	50.5 ± 10.3	41.5 ± 12.9	53.4 ± 11.7	41.1 ± 13.0
Atrial fibrillation at arrival (%)	749 (5.3%)	229 (4.4%)	47 (11.7%)	414 (5.2%)	59 (9.6%)
Atrial fibrillation during admission (%)	1087 (7.7%)	326 (6.3%)	94 (23.4%)	551 (6.9%)	116 (18.9%)
**Coronary angiographic finding**					
Three-vessel disease (%)	1774 (12.5%)	497 (9.6%)	66 (16.4%)	1077(13.4%)	134(21.8%)
Left main disease (%)	803 (5.7%)	202 (3.9%)	44 (10.9%)	507 (6.3%)	50 (8.1%)
Medication at discharge*					
Aspirin (%)	13,030 (95.6%)	5058 (98.1%)	125 (96.9%)	7546 (94.2%)	301 (90.1%)
Clopidogrel (%)	7379 (54.1%)	2123 (41.2%)	94 (72.9%)	4899 (61.2%)	263 (78.7%)
Prasugrel (%)	660 (4.8%)	366 (7.1%)	3 (2.3%)	289 (3.6%)	2 (0.6%)
Ticagrelor (%)	4796 (35.2%)	2548 (49.4%)	27 (20.9%)	2192 (27.4%)	29 (8.7%)
CCB (%)	1712 (12.6%)	274 (5.3%)	9 (7.0%)	1370 (17.1%)	59 (17.7%)
BB (%)	10,837 (79.5%)	4412 (85.6%)	93 (72.1%)	6103 (76.2%)	229 (68.6%)
ACEi (%)	4735 (34.7%)	2082 (40.4%)	43 (33.3%)	2547 (31.8%)	63 (18.9%)
ARB (%)	4948 (36.3%)	1796 (34.8%)	48 (37.2%)	2982 (37.2%)	122 (36.5%)
Statin (%)	12,589 (92.4%)	4869 (94.5%)	112 (86.8%)	7334 (91.5%)	274 (82.0%)
Ezetimide (%)	1338 (9.8%)	576 (11.2%)	3 (2.3%)	747 (9.3%)	12 (3.6%)
Warfarin (%)	203 (1.5%)	71 (1.4%)	3 (2.3%)	120 (1.5%)	9 (2.7%)
NOAC (%)	546 (4.0%)	186 (3.6%)	8 (6.2%)	325 (4.1%)	27 (8.1%)
OHA (%)	3194 (23.4%)	1035 (20.1%)	35 (27.1%)	2007 (25.1%)	117 (35.0%)

Abbreviations: ACEi, angiotensin-converting enzyme inhibitor; AMI, Acute Myocardial Infarction; ARB, angiotensin receptor blocker; BB, beta-blocker; CCB, calcium channel blocker; NOAC, non-vitamin K antagonist oral anticoagulants; OHA, oral hypoglycemic agent; STEMI, ST-segment elevation Myocardial Infarction; NSTEMI, Non-ST-segment elevation Myocardial Infarction.

*The proportion of the medication prescribed at hospital discharge in the death group was calculated after excluding the in-hospital mortality but before excluding patients with missing information during hospital admission: 129 and 334 for the death group in STEMI and NSTEMI, respectively.

### Performance of the predictive models in STEMI

**Figure 1:**
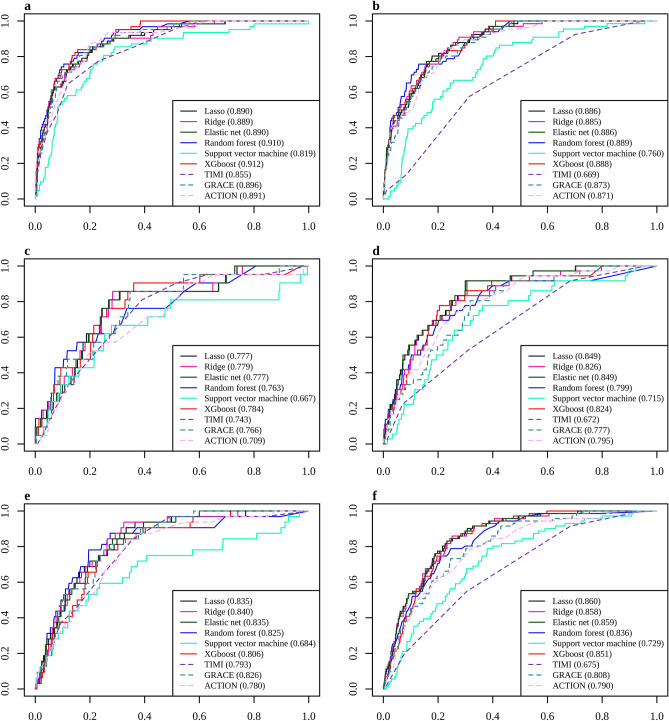
ROC curves of the in-hospital, three-month, and 12-month mortality prediction models in acute myocardial infarction with the traditional predictors. (**a**) In-hospital mortality in STEMI, (**b**) In-hospital mortality in NSTEMI, (**c**) three-month mortality in STEMI, (**d**) three-month mortality in NSTEMI, (**e**) 12-month mortality in STEMI, (**f**) 12-month mortality in NSTEMI.

When the prediction models were built by the ML algorithm using traditional variables in STEMI, the performance was enhanced marginally compared to the best performance among the traditional models (Fig. 1). An evaluation of the performance by the area under the receiver operating characteristic curve (AUC) revealed extreme gradient boosting (XGBoost) to be the best performing model, with an AUC of 0.912 in the ML models, followed by the modified GRACE in the original and modified traditional models (0.901) (Table 2). On the other hand, the other models using the ML algorithms except for the Support Vector Machine (SVM) showed excellent performance over or near the AUC of 0.9. The other traditional models had a lower AUC but were close to 0.9. Regarding the three-month mortality after discharge, the best performing models were XGBoost and GRACE with an AUC of 0.784 and 0.766, respectively, in the ML and traditional models. This was followed in descending order of the AUC by logistic regression regularized with an L2 penalty (Ridge regression), logistic regression regularized with an L1 penalty (Lasso regression), logistic regression regularized with an elastic net penalty (Elastic net), and a Random Forest (RF). For the 12-month mortality, the best performing models were Ridge regression and GRACE in the ML and traditional models, having AUCs of 0.840 and 0.826, respectively. This was followed in descending order of the AUC by Lasso regression and elastic net regression, RF, modified TIMI, and XGBoost. According to the F1-score, the best performing ML model had a score of 0.388, 0.107, and 0.179, respectively, for the in-hospital, three- and 12-month mortality; those were similar or slightly higher than the F1-score of the corresponding traditional models. The highest F1-scores of the modified traditional models were 0.345, 0.075, and 0.170 in predicting the in-hospital, three- and 12-month mortality, respectively.

**Table 2:** Performance in the mortality prediction models in ST-segment elevation myocardial infarction using the traditional features.

	AUC (95% CI)	Specificity	Sensitivity	Accuracy	F1-score
**In-hospital mortality**					
*Machine learning algorithms*					
LR with Lasso	0.890 (0.852–0.928)	0.881	0.726	0.873	0.388
LR with Ridge	0.889 (0.850–0.927)	0.766	0.871	0.772	0.298
LR with Elastic net	0.890 (0.852–0.928)	0.888	0.677	0.876	0.378
RF	0.910 (0.879–0.941)	0.817	0.823	0.817	0.333
SVM	0.819 (0.765–0.873)	0.804	0.677	0.797	0.271
XGBoost	0.912 (0.884–0.939)	0.845	0.839	0.845	0.376
*Traditional and modified traditional model*					
TIMI	0.855 (0.813–0.897)	0.769	0.774	0.769	0.272
GRACE	0.896 (0.862–0.930)	0.842	0.774	0.838	0.347
ACTION-GWTG	0.891 (0.855–0.927)	0.837	0.758	0.832	0.335
Modified TIMI*	0.885 (0.849–0.920)	0.826	0.806	0.825	0.339
Modified GRACE*	0.901 (0.870–0.932)	0.826	0.823	0.826	0.345
Modified ACTION-GWTG*	0.859 (0.810–0.907)	0.833	0.710	0.826	0.312
**3-month mortality**					
*Machine learning algorithms*					
LR with Lasso	0.777 (0.682–0.871)	0.673	0.857	0.677	0.101
LR with Ridge	0.779 (0.683–0.875)	0.620	0.857	0.625	0.088
LR with Elastic net	0.777 (0.683–0.872)	0.652	0.857	0.657	0.095
RF	0.763 (0.656–0.870)	0.801	0.571	0.797	0.107
SVM	0.667 (0.525–0.810)	0.852	0.381	0.842	0.092
XGBoost	0.784 (0.688–0.880)	0.726	0.762	0.727	0.106
*Traditional and modified traditional model*					
TIMI	0.743 (0.650–0.837)	0.610	0.810	0.614	0.082
GRACE	0.766 (0.670–0.862)	0.652	0.857	0.657	0.096
ACTION-GWTG	0.709 (0.602–0.816)	0.630	0.667	0.630	0.070
Modified TIMI*	0.704 (0.593–0.815)	0.628	0.714	0.629	0.075
Modified GRACE*	0.602 (0.458–0.745)	0.832	0.238	0.820	0.053
Modified ACTION-GWTG*	0.653 (0.528–0.778)	0.731	0.476	0.726	0.068
**12-month mortality**					
*Machine learning algorithms*					
LR with Lasso	0.835 (0.776–0.895)	0.799	0.688	0.796	0.179
LR with Ridge	0.840 (0.784–0.896)	0.720	0.844	0.724	0.165
LR with Elastic net	0.835 (0.781–0.889)	0.776	0.719	0.775	0.171
RF	0.825 (0.749–0.901)	0.697	0.875	0.703	0.160
SVM	0.684 (0.574–0.795)	0.592	0.719	0.597	0.103
XGBoost	0.806 (0.743–0.869)	0.782	0.656	0.778	0.160
*Traditional and modified traditional model*					
TIMI	0.793 (0.726–0.860)	0.642	0.844	0.648	0.134
GRACE	0.826 (0.770–0.881)	0.677	0.812	0.681	0.142
ACTION-GWTG	0.780 (0.709–0.850)	0.770	0.562	0.763	0.134
Modified TIMI*	0.802 (0.736–0.868)	0.786	0.688	0.783	0.170
Modified GRACE*	0.741 (0.663–0.820)	0.771	0.625	0.766	0.148
Modified ACTION-GWTG*	0.659 (0.554–0.764)	0.748	0.531	0.741	0.117

AUC, area under the receiver operating characteristic curve; CI, confidential interval; LR, Logistic regression; Lasso, L1 penalty; Ridge, L2 penalty; Elastic net, Elastic net penalty; RF, Random Forest; SVM, Support Vector Machine; XGBoost, Extreme Gradient Boosting; Thrombolysis in myocardial infarction, TIMI; Global registry of acute coronary events, GRACE; Acute coronary treatment and intervention outcomes network—Get With The Guidelines, ACTION-GWTG.

*Traditional models were modified using the recalculated parameters for TIMI, GRACE, and ACTION-GWTG.

### Performance of the predictive models in NSTEMI

The ML models in NSTEMI outperformed the traditional models in predicting the three and 12-month mortality when the ML algorithm was applied to the prediction models, including traditional variables (Table 3). The highest AUCs of the in-hospital mortality prediction models were 0.889 and 0.888 in RF and XGBoost, respectively, which were superior to TIMI (AUC: 0.669) but similar to the modified ACTION-GWTG (AUC: 0.884). For the three-month mortality, the best performing models were Lasso regression (AUC: 0.849) and elastic net regression (AUC: 0.849), which were superior to GRACE (AUC: 0.777) and ACTION-GWTG (AUC: 0.795). The ML models, except for SVM, maintained an AUC > 0.8 for the 12-month mortality, while the AUCs were 0.675 and 0.790 in TIMI and ACTION-GWTG, respectively. The modified GRACE and ACTION-GWTG maintained good performance in predicting the 12-month mortality in addition to GRACE. Based on the F1-score, the best performing ML models were Lasso regression, elastic net, and XGBoost with a score of 0.236, 0.130, and 0.225 for the in-hospital, three- and 12-month mortality, respectively, while the highest figures were 0.224, 0.114, and 0.196, respectively in the traditional models. For the modified traditional models, the highest F1-scores were 0.243, 0.110, and 0.206 in predicting the in-hospital, three- and 12-month mortality, respectively.

**Table 3:** Performance of the mortality prediction models in Non-ST-segment elevation myocardial infarction using the traditional features.

	AUC (95% CI)	Specificity	Sensitivity	Accuracy	F1-score
**In-hospital mortality**					
*Machine learning algorithms*					
LR with Lasso	0.886 (0.855–0.918)	0.793	0.818	0.794	0.236
LR with Ridge	0.885 (0.852–0.918)	0.810	0.758	0.808	0.235
LR with Elastic net	0.886 (0.854–0.918)	0.791	0.803	0.792	0.230
RF	0.889 (0.856–0.923)	0.793	0.758	0.792	0.220
SVM	0.760 (0.707–0.813)	0.709	0.667	0.707	0.150
XGBoost	0.888 (0.857–0.919)	0.785	0.803	0.786	0.226
*Traditional and modified traditional model*					
TIMI	0.669 (0.613–0.724)	0.686	0.576	0.682	0.123
GRACE	0.873 (0.840–0.906)	0.734	0.803	0.736	0.191
ACTION-GWTG	0.871 (0.836–0.907)	0.812	0.712	0.808	0.224
Modified TIMI*	0.709 (0.656–0.763)	0.506	0.788	0.516	0.112
Modified GRACE*	0.876 (0.841–0.912)	0.806	0.773	0.805	0.235
Modified ACTION-GWTG*	0.884 (0.851–0.916)	0.819	0.758	0.817	0.243
**3 month mortality**					
*Machine learning algorithms*					
LR with Lasso	0.849 (0.795–0.903)	0.728	0.833	0.731	0.127
LR with Ridge	0.826 (0.764–0.889)	0.719	0.833	0.722	0.124
LR with Elastic net	0.849 (0.795–0.904)	0.735	0.833	0.738	0.130
RF	0.799 (0.719–0.878)	0.681	0.778	0.683	0.104
SVM	0.715 (0.633–0.798)	0.557	0.778	0.562	0.077
XGBoost	0.824 (0.760–0.888)	0.654	0.861	0.659	0.106
*Traditional and modified traditional model*					
TIMI	0.672 (0.592–0.751)	0.689	0.528	0.685	0.073
GRACE	0.777 (0.711–0.844)	0.705	0.694	0.704	0.100
ACTION-GWTG	0.795 (0.728–0.862)	0.726	0.750	0.727	0.114
Modified TIMI*	0.675 (0.596–0.754)	0.534	0.750	0.539	0.071
Modified GRACE*	0.774 (0.709–0.838)	0.623	0.778	0.627	0.089
Modified ACTION-GWTG*	0.782 (0.721–0.843)	0.759	0.639	0.756	0.110
**12 month mortality**					
*Machine learning algorithms*					
LR with Lasso	0.860 (0.825–0.895)	0.693	0.901	0.703	0.219
LR with Ridge	0.858 (0.821–0.894)	0.710	0.859	0.717	0.219
LR with Elastic net	0.859 (0.824–0.894)	0.721	0.845	0.727	0.222
RF	0.836 (0.796–0.876)	0.688	0.803	0.694	0.195
SVM	0.729 (0.675–0.784)	0.625	0.746	0.631	0.157
XGBoost	0.851 (0.817–0.884)	0.725	0.845	0.731	0.225
*Traditional and modified traditional model*					
TIMI	0.675 (0.619–0.731)	0.695	0.549	0.688	0.140
GRACE	0.808 (0.764–0.852)	0.697	0.789	0.701	0.196
ACTION-GWTG	0.790 (0.740–0.839)	0.719	0.718	0.719	0.191
Modified TIMI*	0.729 (0.683–0.776)	0.545	0.845	0.559	0.150
Modified GRACE*	0.820 (0.779–0.861)	0.715	0.761	0.717	0.199
Modified ACTION-GWTG*	0.808 (0.768–0.848)	0.739	0.732	0.739	0.206

AUC, area under the receiver operating characteristic curve; LR, Logistic regression; Lasso, L1 penalty; Ridge, L2 penalty; Elastic net, Elastic net penalty; RF, Random Forest; SVM, Support Vector Machine; XGBoost, Extreme Gradient Boosting; Thrombolysis in myocardial infarction, TIMI; Global registry of acute coronary events, GRACE; Acute coronary treatment and intervention outcomes network—Get With The Guidelines, ACTION-GWTG.

*Traditional models were modified using the recalculated parameters for TIMI, GRACE, and ACTION-GWTG.

### Comparison of the performance between the ML and traditional models

A comparison of all the ML models with three conventional models according to the statistical significance revealed the ML models to be superior to the traditional models in predicting the long-term mortality in NSTEMI (Supplementary Table 2). The ML models outperformed TIMI in predicting the in-hospital mortality among the NSTEMI patients, while they were similar to GRACE and ACTION-GWTG. On the other hand, Lasso and elastic net regression were superior to all three traditional models in predicting the three-month mortality for those who survived to discharge. Moreover, Lasso, Ridge, and elastic net regression, and XGBoost had significantly higher AUCs in predicting the 12-month mortality than TIMI, GRACE, and ACTION-GWTG. In contrast, with STEMI, RF and XGBoost were the only ML models that significantly outperformed TIMI in predicting the in-hospital mortality. Otherwise, the difference between the traditional and all the ML models was not statistically significant. A comparison of the ML models with the modified traditional models revealed consistent findings (Supplementary Table 3). The differences between the ML models and the modified traditional models were statistically significant among AMI patients, particularly in predicting long-term mortality.

### Effect of optional clinical features and medication at discharge

**Figure 2:**
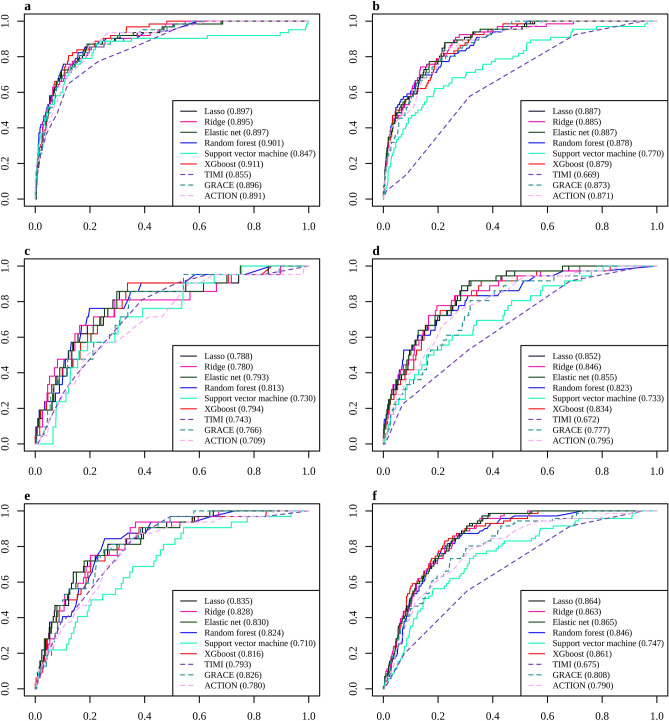
ROC curves of the in-hospital, three-month, and 12-month mortality prediction models in acute myocardial infarction with traditional and optional predictors. (**a**) In-hospital mortality in STEMI, (**b**) In-hospital mortality in NSTEMI, (**c**) 3-month mortality in STEMI, (**d**) 3-month mortality in NSTEMI, (**e**) 12-month mortality in STEMI, (**f**) 12-month mortality in NSTEMI.

The performance was not enhanced by including the optional predictors in the models (Fig. 2). The highest AUC was 0.911 for XGBoost in the ML model, including the optional predictors in STEMI, which was similar to the 0.912 for XGBoost, including the traditional predictors only (Supplementary Table 4). In the case of the three-month mortality in STEMI, the highest AUC was 0.813 for RF, including all predictors, which was similar to the 0.784 for XGBoost, including the traditional predictors only. For the 12-month mortality, the figures were 0.835 in Lasso regression, including all the predictors, and 0.840 in Ridge regression, including the traditional predictors only. With NSTEMI (Supplementary Table 5), the best performing ML models reached 0.887, 0.855, and 0.865 for in-hospital, three-month, and 12-month mortality, respectively, in the ML model including all the predictors, whereas the corresponding numbers were 0.889, 0.849, and 0.860 in the ML model including the traditional predictors. None of the ML models, except for the SVM, showed a significant difference in the AUCs when the performance of the models with the traditional predictors only was compared with the model applying all the predictors. Moreover, a comparison of the ML models, including the traditional and optional predictors, and the corresponding models, including medication at discharge, showed no significant difference in both STEMI and NSTEMI (Supplementary Table 6).

Supplementary Tables 7 and 8 list the importance of the variables. The variable importance was different for each prediction model. Some variables in the traditional predictors in the ML models were excluded, whereas some of the optional variables were included. Furthermore, the performance of the predictive models did not change significantly in both STEMI and NSTEMI when the problem of class imbalance was addressed (Supplementary Table 9 and 10). The highest AUC of the ML models was similar to that of the models using up-sampling, down-sampling, and SMOTE. Only the SVM benefited from balancing the classification using re-balancing methods.

### Performance in external validation

The performance of the ML-based models was validated externally using the Korean Acute Myocardial Infarction Registry-National Institutes of Health (KAMIR-NIH) database, which is an independent prospective multicenter registry (Table 4). The AUCs exceeded 0.9 except for the SVM for in-hospital mortality among the patients with STEMI and NSTEMI, but those were close to 0.8 for the 12-month mortality. The ML models were superior to the traditional model in predicting the 12-month mortality in NSTEMI, which is similar to the finding using the test data. On the other hand, the F1 scores in the KAMIR-NIH registry were lower than those in the internal validation.

**Table 4:** Performance of the mortality prediction models tested in the Korean Acute Myocardial Infarction Registry-National Institutes of Health.

	STEMI					NSTEMI				
	AUC (95% CI)	Specificity	Sensitivity	Accuracy	F1-score	AUC (95% CI)	Specificity	Sensitivity	Accuracy	F1-score
**In-hospital mortality**						**In-hospital mortality**				
*Machine learning algorithms*						*Machine learning algorithms*				
LR with Lasso	0.923 (0.897–0.948)	0.877	0.807	0.876	0.124	0.916 (0.891–0.941)	0.848	0.787	0.847	0.096
LR with Ridge	0.923 (0.898–0.948)	0.755	0.982	0.757	0.081	0.918 (0.894–0.942)	0.868	0.770	0.867	0.107
LR with Elastic net	0.923 (0.898–0.948)	0.884	0.754	0.883	0.123	0.917 (0.893–0.941)	0.845	0.803	0.845	0.096
RF	0.924 (0.897–0.952)	0.815	0.860	0.816	0.092	0.924 (0.903–0.946)	0.860	0.803	0.860	0.106
SVM	0.875 (0.844–0.907)	0.772	0.807	0.773	0.072	0.848 (0.815–0.880)	0.723	0.852	0.725	0.060
XGBoost	0.938 (0.920–0.955)	0.855	0.860	0.855	0.114	0.911 (0.885–0.937)	0.832	0.787	0.832	0.088
*Traditional model*						*Traditional model*				
TIMI	0.866 (0.820–0.913)	0.774	0.807	0.774	0.072	0.672 (0.612–0.731)	0.693	0.590	0.692	0.038
GRACE	0.921 (0.891–0.950)	0.851	0.825	0.850	0.107	0.917 (0.890–0.944)	0.799	0.852	0.800	0.081
**12-month mortality**						**12-month mortality**				
*Machine learning algorithms*						*Machine learning algorithms*				
LR with Lasso	0.789 (0.719–0.860)	0.751	0.696	0.750	0.048	0.815 (0.781–0.848)	0.727	0.720	0.726	0.100
LR with Ridge	0.789 (0.718–0.859)	0.636	0.761	0.637	0.037	0.809 (0.774–0.843)	0.735	0.695	0.735	0.099
LR with Elastic net	0.789 (0.721–0.858)	0.721	0.696	0.721	0.044	0.814 (0.780–0.847)	0.749	0.695	0.748	0.104
RF	0.772 (0.702–0.843)	0.572	0.826	0.575	0.034	0.792 (0.751–0.832)	0.746	0.703	0.745	0.104
SVM	0.687 (0.606–0.768)	0.425	0.804	0.429	0.025	0.721 (0.676–0.765)	0.662	0.695	0.663	0.080
XGBoost	0.796 (0.736–0.857)	0.701	0.717	0.701	0.042	0.808 (0.773–0.843)	0.783	0.653	0.781	0.111
*Traditional model*						*Traditional model*				
TIMI	0.701 (0.633–0.769)	0.624	0.804	0.626	0.038	0.676 (0.635–0.717)	0.693	0.590	0.692	0.038
GRACE	0.738 (0.671–0.806)	0.650	0.761	0.651	0.038	0.778 (0.741–0.814)	0.799	0.852	0.800	0.081

AUC, area under the receiver operating characteristic curve; LR, Logistic regression; Lasso, L1 penalty; Ridge, L2 penalty; Elastic net, Elastic net penalty; RF, Random Forest; SVM, Support Vector Machine; XGBoost, Extreme Gradient Boosting.

## Discussion

Mortality prediction models were developed using several ML algorithms (Lasso regression, Ridge regression, elastic net, RF, SVM, and XGBoost). Their performance was comparable in predicting the short- and long-term mortality of patients with STEMI with those of traditional risk stratification with comparable predictors. On the other hand, the discrimination improved the existing the prognosis prediction tools in NSTEMI, particularly in predicting long-term mortality. Furthermore, adding more clinical variables to the models did not enhance the performance of the predictive models for mortality in AMI.

The ML algorithms outperformed the traditional risk score methods when the predictors were the same, but the difference was similar in STEMI, and the best working algorithms varied according to the predictors and outcomes. Some studies suggested applying ML algorithms to enhance the performance of the prognosis prediction model for patients with AMI^11,17^. A recent study reported that deep learning (AUC: 0.905) could outperform the GRACE score (AUC: 0.851) in predicting the in-hospital mortality of AMI patients^11^. The other study suggested that when predicting cardiac and sudden death during a one-year follow-up, the AUC in the ML models was improved by 0.08 compared to that in GRACE^17^. Another study reported AUCs of 0.828, 0.895, 0.810, and 0.882 in an artificial neural network (ANN), decision tree (DT), naïve Bayes (NB), and SVM, respectively, for the 30-day mortality, which were slightly higher than or similar to the values (0.83) from the GRACE risk score methods suggested in the validation study^3,18^. On the other hand, the previous study did not compare the performance between the conventional models and the ML models in the research data, so that it could only be inferred indirectly^18^. Although the above three studies showed that ML algorithms could enhance discrimination, other researchers proposed that the ML models were not always preferable to the traditional model. Some studies on the prognosis of AMI patients suggested that ML models were not superior but showed comparable performance to the regression-based approach^19–21^. One study using the administrative database of the National Inpatient Sample showed that RF (AUC: 0.85) was comparable to the traditional LR (AUC: 0.84) in predicting the in-hospital mortality among women with STEMI^19^. Another study showed that the best performance of ML models was similar to that of the GRACE score (AUC: 0.91 vs. 0.87)^20^. Austen et al. reported that when the cubic spline was included in the LR, it outperformed the ML models of the RF, regression trees (RT), bagged RT, and boosted RT^21^.

This study showed that ML models were better than the traditional models in NSTEMI but could not reach statistical significance in STEMI. The different superiority of the ML models compared to the traditional models in STEMI and NSTEMI may partially explain the inconsistency of the literature^11,17–20^. Two of the three studies showing comparable performance between the traditional and ML models included patients with STEMI only^19,20^. In contrast, all three studies showing superior performance of the ML models included all patients with STEMI and NSTEMI^11,17,18^. Although this could not explain all the inconsistency because subgroup analysis showed that ML also outperformed GRACE in STEMI in a previous study^11^, the different performances of the ML models between the STEMI and NSTEMI groups may have contributed to the inconsistent findings. The ML models may have higher discrimination in the NSTEMI group than the traditional model because NSTEMI has more heterogeneous clinical and pathological features than STEMI^22,23^. STEMI results from a complete thrombotic occlusion of the infarct-related artery, while NSTEMI occurs in more heterogeneous conditions, such as incomplete coronary occlusion, coronary artery spasm, coronary embolism, myocarditis, and others^24^. Moreover, ML-based models could outperform the traditional models when analyzing complex data because of the non-parametric assumption, non-linearity, and higher-order interaction. Furthermore, the inconsistency appears to be due to the relatively small difference in the AUC between the ML model and GRACE because the GRACE risk score was updated in 2014, and the continuous variables were divided into many categories to reflect the non-linear relationship^8^. The ML-based models also require tuning parameters that may influence the model performance, which may fit and perform differently in different datasets^14^.

Traditional risk stratification focused on predicting the short-term mortality, while only a few suggested the one-year mortality. The CADILLAC risk score developed in 2005 showed good performance for the one-year mortality (c-statistic of 0.79). Moreover, GRACE 2.0, which was updated in 2014 considering the non-linear relationship between mortality and continuous variables, showed an AUC of 0.82^3,8,25^. After introducing the ML algorithms, some studies suggested that discrimination could be improved to predict the long-term mortality^15,17^. One recent study on the one-year mortality showed that the AUC of the prediction model could be up to 0.901 among patients admitted to the ICU with AMI, which was achieved using the Logistic Model Trees^15^. Another study also showed good discriminative power for the one-year mortality with an AUC of 0.898, which was achieved using either the Deep Neural Network or Gradient Boosting Machine^17^. The present study suggested that ML models maintained good discrimination for the 12-month mortality, but the AUC value was lower than those of the two previous studies^15,17^. This might be because the one-year mortality was defined not as the cumulative mortality, including in-hospital mortality, as in other studies, but as the mortality of those who survived at hospital discharge during the one-year follow-up. The current study aimed to help cardiologists make a treatment and management plan considering the risk of mortality when a patient is discharged.

This study showed that the performance of the prediction model was not increased significantly by adding the optional variables. This might be because the optional variables used in this study could not add more information to the ML models in predicting the mortality of patients with AMI. Only a few studies revealed the influence of features on the performance of prediction models. One study on the prediction model of the 30-day mortality after STEMI showed that the performance of most ML algorithms plateaued when the models introduced the highest 15 ranked variables among 54 variables^20^. Another study on the one-year mortality of patients with anterior STEMI showed a change in the performance of the prediction model when the top 20 ranked variables were selected instead of all 59 variables^26^. For RF, the AUC barely changed from 0.932 in the full model to 0.944 with the 20 features, while the changes depended on the model. The AUC decreased from 0.931 to 0.864 in LR, while it increased from 0.772 to 0.852 in the decision tree. The top 20 variables listed in their study were as follows: New York Heart Association Classification at discharge, heart failure at admission, heart rate, age, left ventricular ejection fraction, serum cystatin, initial BNP, platelet count, fibrinogen, serum creatinine, blood glucose, systolic blood pressure, diastolic blood pressure, total bilirubin, blood urea nitrogen, and revascularization type. Only five variables overlapped with the traditional variables in the present study. The predictive models using the ML algorithm appeared to be less dependent on the specific predictors because many clinical predictors influenced and reflected one another. ML algorithms, which allow non-linearity, higher-order effects, and interactions, may not depend on specific predictors as much as the traditional risk stratification methods.

This study suggested that the ML algorithm could enhance the performance of predictive models in AMI and pointed out the particular area where the predictive models could benefit from applying ML algorithms in AMI. Hence, clinicians can identify better those at high risk of mortality in NSTEMI using ML prediction models and focus on the high-risk group at admission and discharge. The ML-based prediction model could be integrated into the electronic medical records as a part of clinical decision support and be utilized in clinical practice. This model will inform clinicians of those who require close monitoring and intensive care during the hospital stay and require frequent follow-up and high medication adherence at discharge.

This study had some limitations. First, the ML algorithm is less intuitive than the risk scoring system developed using traditional statistical analysis. The prediction model developed using the ML algorithm. The importance of predictors in the model is more challenging to interpret because they could contain non-linear models and ensemble methods. Moreover, the proposed prediction model may be specific to the study population, Korean patients with AMI. A previous study reported different risk factors and responses to medical and interventional treatments between Korean and Western AMI patients. Hence, predictive models could show different performance measures in other populations, and ML algorithms should be compared to confirm which is best^27,28^. Despite the improvement of AUC, the F1 scores were low in both the ML and traditional models, and the difference in the F1 scores between the ML and traditional models was small. Moreover, the statistical difference in the F1 scores could not be evaluated. Ranganathan and Aggarwal demonstrated it with an example that a test with good sensitivity and specificity could have low precision when applied to a disease with a low pretest probability^29^. The low F1 score in the current study may be due to the low precision and low mortality rate. They suggested that it would be prudent to apply a diagnostic test only in those with a high pretest probability of the disease^29^, and it could be interpreted that the F1-score would increase if it is applied to patients with moderate to high severity. Future research should set a proper indication of the mortality prediction model or enhance the precision and F1 score for all patients with AMI.

## Conclusion

A prediction model for short- and long-term mortality was generated in patients admitted with AMI using multicenter registries and validated using independent cohort data. The ML-based approach increased the discriminative performance of the patients with NSTEMI in predicting mortality compared to the traditional risk scoring method. On the other hand, the performance did not depend on the inclusion of more predictors.

## Methods

### Data source

A retrospective cohort study was conducted using the data from the Korean Registry of Acute Myocardial Infarction for Regional Cardiocerebrovascular Centers (KRAMI-RCC) registry. The KRAMI-RCC is a prospective multicenter registry of AMI in Korea. The data were collected from all 14 Regional Cardiocerebrovascular Centers (RCCVCs) established by the Ministry of Health and Welfare for the prevention and treatment of cardiovascular disease in Korea since 2008. The purpose and impact of RCCs on AMI are published elsewhere^30,31^. KRAMI-RCC is a web-based registry of consecutive AMI cases reflecting real-world information on the clinical practice in RCCs and consists of pre-hospital, hospital, and post-hospital data. The institutional review board of Inha University Hospital approved this study protocol, and the need for informed consent was waived because of the retrospective nature of the study using anonymized data with minimal potential for harm (IRB number: 2020–05-035). All methods were carried out in accordance with the relevant guidelines and regulations, and the data were obtained with the approval of the committee of RCCVCs after anonymization.

### Study participants

**Figure 3:**
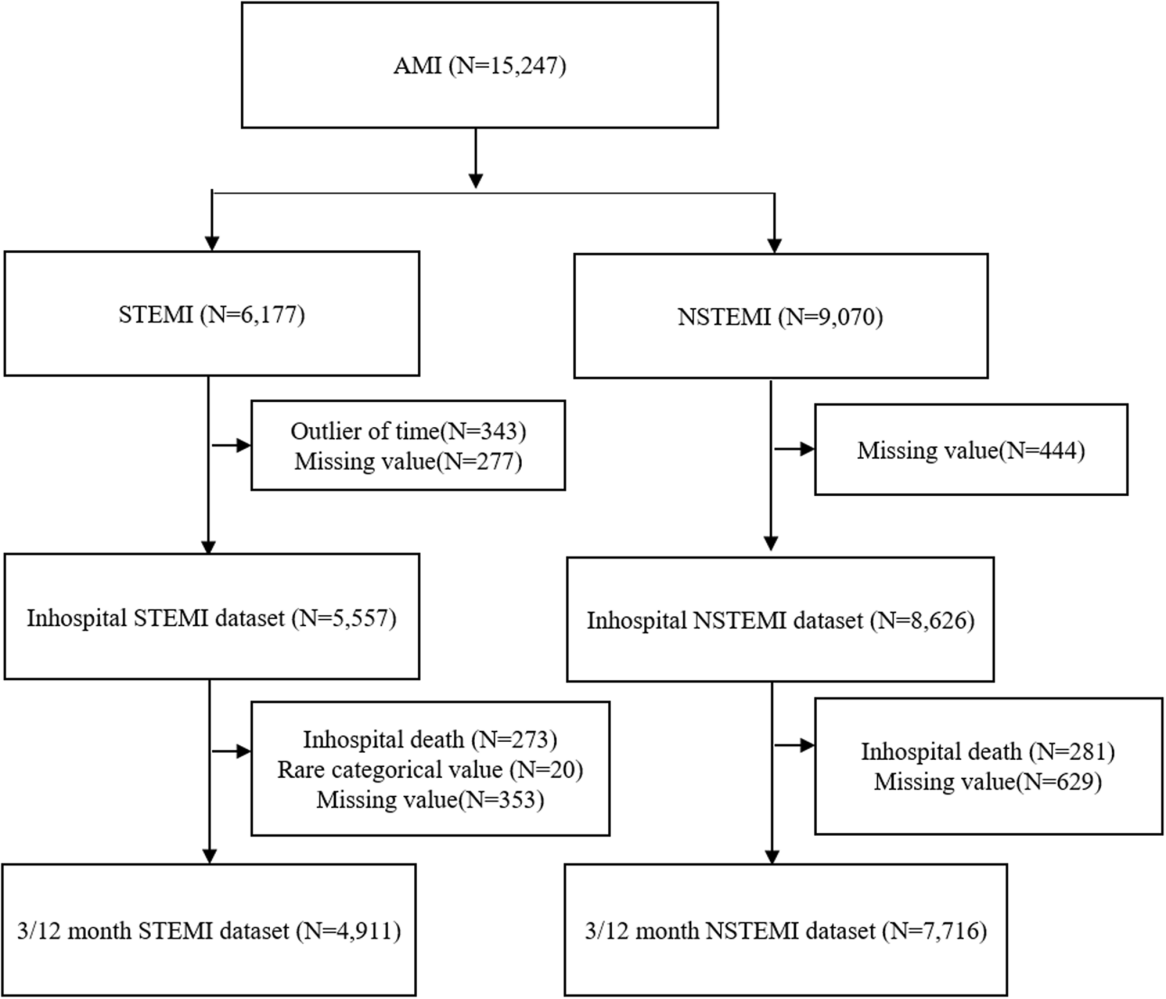
Flowchart of study inclusion. AMI, Acute Myocardial Infarction; STEMI, ST-segment elevation Myocardial Infarction; NSTEMI, Non-ST-segment elevation Myocardial Infarction.

All enrolled participants were patients diagnosed with AMI and admitted to the RCCs through the emergency department (ED). This study included 15,247 patients with AMI in KRAMI-RCC from July 2016 to July 2019 who finished the 12-month follow up in this research. The exclusion criteria were (1) less than 18 years of age, (2) chest pain onset more than 24 h in STEMI, and (3) missing data to calculate the traditional risk score: TIMI, GRACE, and ACTION-GWTG. Of the 15,247 patients enrolled in the KRAMI registry, 6177 and 9070 patients were diagnosed with STEMI and NSTEMI, respectively (Fig. 3). After excluding patients with missing data on the predictors at the emergency department (ED) or before ED arrival and those who visited the hospital 24 h after symptom onset, 5557 patients with STEMI were eligible for the final analysis of in-hospital mortality. Furthermore, patients who survived upon discharge were included in the final analysis of the three- and 12-month mortality. This study excluded missing data on the clinical predictors during hospital admission and rare categorical responses among the survivors at hospital discharge. Therefore, the number of patients with STEMI was 4911 for the final analysis of the three and 12-month mortality. For NSTEMI, the number of patients was 8626 for a final analysis of the in-hospital mortality after excluding missing data at the pre-ED or ED level. Regarding the three and 12-month mortality, the number of patients with NSTEMI was 7716 after excluding missing data during the hospital stay and in-hospital deaths.

### Predictors

The possible predictors for mortality were extracted from the database based on previous studies, including demographic information, past medical history, initial symptoms, laboratory findings, events before ED arrival and during the hospital stay, and coronary angiographic findings^3,4,6,8–10^. The predictors were classified according to the time frame (pre-ED, ED, and hospital admission). The predictors used in the traditional risk stratification model were selected as the traditional variables^3^; the other predictors were categorized as optional variables, as described in Supplementary Table 11. The predictors for in-hospital mortality were limited to the variables available in the pre-ED and ED stage. In contrast, those for the three-month and 12-month mortality included all the variables in the pre-ED, ED, and hospital admission stage. Furthermore, medication at discharge was also included in the model for predicting the three- and 12-month mortality.

### Outcomes

The outcomes of interest in this study were in-hospital, three-month, and 12-month mortality. The patients who survived to discharge were followed up by telephone at three and 12 months. The follow-up information was collected through contact with the patients or their families. If unavailable, a follow-up visit or death certificate on the electronic medical records was also checked to determine death.

### Predictive models

ML algorithms, such as RF, SMV, XGBoost, Lasso, Ridge regression, and Elastic net, were applied to develop a mortality prediction model. RF builds multiple decision trees and merges them to make a more accurate and stable prediction, while XGBoost provides a parallel tree boosting with a gradient descent that solves many data science problems in a fast and accurate manner. SVM constructs a hyperplane or a set of hyperplanes in high- or infinite-dimensional space for classification.

For each prediction model, tenfold cross-validation was used to tune the hyperparameters, with the AUC as the evaluation standard. The hyperparameters in RF were tuned by searching for all the combinations of the number of trees (500, 1000, and 2000) and the number of variables (2, 4, 6, and 8). For XGboost, this study searched for all the combinations of the number of boosting iterations (25, 50, 75, 100, 125, and 150), learning rate (0.05, 0.1, and 0.3), minimum loss reduction (0 and 5), and the maximum depth of the tree (4, 6, and 8). Regarding SVM, the hyperparameters were optimized with combinations of the cost of constraints violation (0.0039, 0.0625, 1.0000, and 2.0000) and bandwidth of the radial kernel (0.0039, 0.0625, 1.0000, and 2.0000). For Lasso, Ridge regression, and elastic net, the default setting of ‘glmnet’ package in R was used to select the hyperparameters^32^.

Three different sampling methods were also considered to adjust the highly imbalanced classes: up-sampling, down-sampling, and synthetic minority oversampling technique (SMOTE). The number of study participants in the training set changed from 4443 to 8464, 422, and 1477 when up-sampling, down-sampling, and SMOTE, respectively, were applied to the in-hospital mortality data of STEMI. The number of participants was 13,422, 430, and 1505 in the datasets of up-sampling, down-sampling, and SMOTE for the in-hospital mortality data of NSTEMI.

### Traditional and modified traditional models

TIMI and the updated version of GRACE and ACTION-GWTG were used as the references of the traditional models to compare with ML^3,4,8,13,33^. The TIMI risk scores for STEMI and NSTEMI were used in this study^4,33^. The TIMI for STEMI and NSTEMI was developed to predict the 30-day and 14-day mortality, respectively, whereas the prognostic capacity of TIMI for STEMI was stable over multiple time points from 24 h to one year after hospital admission^4^. GRACE v2.0, in which Anderson et al. updated the initial GRACE risk score in 2014, used non-linear functions to enhance discrimination^8^. Although it was developed to predict the six-month mortality, it was validated externally over the longer term with an AUC of 0.82 at one and three-year mortality. In another validation study, GRACE v2.0 also showed excellent discrimination with an AUC of 0.91 for predicting the in-hospital mortality^34^. The updated ACTION-GWTG developed in 2016 had high discrimination with an AUC of 0.88 to predict in-hospital mortality^13^.

These traditional models were fitted to the training data and modified by recalculating the model parameters. In addition to the original traditional models, the modified traditional model was compared with the ML models.

### Analysis and Performance measures

The continuous variables, such as age and weight, are represented as the mean and standard deviation in statistical analysis, while the categorical variables are the frequency and proportion. After standardization, the data were split by random sampling into a training set (80%) for developing the ML-based models and a test set (20%) for internal validation. The performance of the mortality prediction model was evaluated using the test data, and was described by the sensitivity, specificity, accuracy, F1-score, and area under the receiver operating characteristic curves (AUC) in the tables and the receiver operating characteristics (ROC) curve in the plots. The AUC of the ML algorithms was suggested with a 95% confidence interval and was compared with traditional risk stratification (TIMI, GRACE, and ACTION-GWTG) using a DeLong Test^35^. All analyses were implemented using R software version 4.0.0 (R Development Core Team, Vienna, Austria)^36^.

### Validation

In addition to internal validation using a test set, external validation was performed using the KAMIR-NIH registry, which is a prospective multicenter registry in Korea. The registry enrolled patients diagnosed with AMI at 20 tertiary university hospitals who were eligible for primary PCI from November 2011 to December 2015. The detailed study protocols are published elsewhere^37^. The performance of the ACTION-GWTG was not estimated because prior peripheral arterial disease was not collected in the KAMIR-NIH registry. Moreover, the three-month mortality was not available due to different follow-up schedules in the registry. The ML models were validated for the in-hospital and 12-month mortality after matching the operational definition of the pre-ED cardiac arrest and abnormal cardiac biomarkers.

## Supplementary Information

Supplementary Information.

## Data Availability

The data that support the findings of this study are available from KRAMI-RCC, but restrictions apply to the availability of these data. Data are available from the authors upon reasonable request and with permission of KRAMI-RCC.

## Abbreviations

AMI: Acute myocardial infarction
STEMI: ST-segment elevation myocardial infarction
NSTEMI: Non-ST segment elevation myocardial infarction
ML: Machine learning
TM: Traditional model
AUC: Areas under the receiver operating characteristic curve
RF: Random forest
SVM: Support vector machine
XGBoost: Extreme Gradient Boosting
Lasso: Logistic regression regularized with L1 penalty
Ridge regression: Logistic regression regularized with L2 penalty
Elastic net: Logistic regression regularized with an elastic net penalty
RCCVC: Regional Cardiocerebrovascular Center
KRAMI-RCC: The Korean Registry of Acute Myocardial Infarction for Regional Cardiocerebrovascular Centers
KAMIR-NIH: Korean Acute Myocardial Infarction Registry-National Institutes of Health
