# Supplementary material for: Machine learning enhances the performance of short and long-term mortality prediction model in non-ST-segment elevation myocardial infarction
Source: Sci Rep. 2021 Jun 18;11:12886. doi: 10.1038/s41598-021-92362-1 (PMC8213755; doi:10.1038/s41598-021-92362-1)
Supplement: Supplementary file 1 — Supplementary Information. [file 41598_2021_92362_MOESM1_ESM.docx]

Supplementary information

**Machine learning enhances the performance of short and long-term mortality prediction model in non-ST-segment elevation myocardial infarction**

Woojoo Lee^1^, Joongyub Lee^2^, Seoung-Il Woo^3^, Seong Huan Choi^3^, Jang-Whan Bae^4^, Seungpil Jung^1^, Myung Ho Jeong^5^, Won Kyung Lee^6*^

^1^Department of Public Health Sciences, Graduate School of Public Health, Seoul National University

^2^Department of Preventive Medicine, Seoul National University College of Medicine

^3^Department of Cardiology, Inha University Hospital, School of Medicine, Inha University

^4^Division of Cardiology, Department of Internal Medicine, Chungbuk National University College of Medicine

^5^ Chonnam National University Hospital, Gwangju, Republic of Korea

^6^Department of Prevention and Management, Inha University Hospital, School of Medicine, Inha University, Republic of Korea

*Corresponding author

27 Inhang-Ro, Jung-Gu, Inha Univerisity Hospital, Incheon, Republic of Korea

Telephone: +82-32-890-2885, Mobile: +82-10-6360-7965, Fax: +82-32-890-3459

E-mail: bluewhale65@inha.ac.kr

Supplementary Table 1. Number of participants with survival and death in the dataset of the in-hospital, three-month, and 12-month mortality after excluding missing information on the predictors

| Data | Mortality | KRAMI dataset (100%) | | | Training set (80%) | | | Test set (20%) | | | External validation set | | |
| --- | --- | --- | --- | --- | --- | --- | --- | --- | --- | --- | --- | --- | --- |
|  |  | Total | Survival | Death | Total | Survival | Death | Total | Survival | Death | Total | Survival | Death |
| STEMI | In hospital | 5557 | 5284 | 273 | 4443 | 4232 | 211 | 1114 | 1052 | 62 | 5235 | 5178 | 57 |
|  | 3 months | 4911 | 4843 | 68 | 3918 | 3871 | 47 | 993 | 972 | 21 | - | - | - |
|  | 12 months | 4911 | 4791 | 120 | 3922 | 3834 | 88 | 989 | 957 | 32 | 5031 | 4985 | 46 |
| NSTEMI | In hospital | 8626 | 8345 | 281 | 6926 | 6711 | 215 | 1700 | 1634 | 66 | 5924 | 5863 | 61 |
|  | 3 months | 7716 | 7574 | 142 | 6187 | 6081 | 106 | 1529 | 1493 | 36 | - | - | - |
|  | 12 months | 7716 | 7410 | 306 | 6178 | 5943 | 235 | 1538 | 1467 | 71 | 5622 | 5504 | 118 |

Supplementary Table 2. Pairwise comparison of the area under the receiver operating characteristic curve between machine learning models and traditional models using a DeLong Test

| ***p-value*** | STEMI | | | NSTEMI | | |
| --- | --- | --- | --- | --- | --- | --- |
|  | TIMI | GRACE | ACTION | TIMI | GRACE | ACTION |
| **In-hospital** |  |  |  |  |  |  |
| Lasso | 0.0886 | 0.5844 | 0.9543 | <0.0001 | 0.1348 | 0.2206 |
| Ridge | 0.0996 | 0.5234 | 0.8924 | <0.0001 | 0.2050 | 0.2743 |
| Elastic net | 0.0864 | 0.6061 | 0.9709 | <0.0001 | 0.1412 | 0.2222 |
| RF | 0.0057 | 0.3121 | 0.2378 | <0.0001 | 0.2579 | 0.2817 |
| SVM | 0.2090 | 0.0028 | 0.0089 | 0.0276 | <0.0001 | <0.0001 |
| XGBoost | 0.0031 | 0.1626 | 0.1304 | <0.0001 | 0.1872 | 0.2879 |
| **3 month** |  |  |  |  |  |  |
| Lasso | 0.5021 | 0.8381 | 0.2848 | <0.0001 | 0.0041 | 0.0211 |
| Ridge | 0.4006 | 0.7497 | 0.2160 | <0.0001 | 0.0642 | 0.1702 |
| Elastic net | 0.4376 | 0.7946 | 0.2515 | <0.0001 | 0.0044 | 0.0212 |
| RF | 0.5443 | 0.9342 | 0.2881 | 0.0032 | 0.5312 | 0.9091 |
| SVM | 0.3087 | 0.2168 | 0.6568 | 0.3690 | 0.0793 | 0.0186 |
| XGBoost | 0.1886 | 0.6167 | 0.1505 | <0.0001 | 0.0918 | 0.2993 |
| **12 month** |  |  |  |  |  |  |
| Lasso | 0.2545 | 0.7033 | 0.1481 | <0.0001 | 0.0052 | 0.0008 |
| Ridge | 0.2147 | 0.6313 | 0.1398 | <0.0001 | 0.0086 | 0.0011 |
| Elastic net | 0.2497 | 0.7218 | 0.1366 | <0.0001 | 0.0061 | 0.0009 |
| RF | 0.3544 | 0.9813 | 0.2631 | <0.0001 | 0.2083 | 0.0713 |
| SVM | 0.0221 | 0.0021 | 0.0867 | 0.1541 | 0.0142 | 0.0848 |
| XGBoost | 0.7450 | 0.5078 | 0.4901 | <0.0001 | 0.0255 | 0.0042 |

Lasso, Logistic regression with L1 penalty; Ridge, Logistic regression with L2 penalty; Elastic net, Logistic regression with Elastic net penalty; RF, Random Forest; SVM, Support Vector Machine; XGBoost, Extreme Gradient Boosting; TIMI; Global registry of acute coronary events, GRACE; Acute coronary treatment and intervention outcomes network – Get With The Guidelines, ACTION-GWTG

Supplementary Table 3. Pairwise comparison of area under the receiver operating characteristic curve between machine learning models and modified traditional models using the DeLong Test

| ***p-value*** | STEMI | | | NSTEMI | | |
| --- | --- | --- | --- | --- | --- | --- |
|  | Modified TIMI* | Modified GRACE* | Modified ACTION* | Modified TIMI* | Modified GRACE* | Modified ACTION* |
| **In-hospital** |  |  |  |  |  |  |
| Lasso | 0.7338 | 0.4161 | 0.1312 | <0.0001 | 0.4333 | 0.8200 |
| Ridge | 0.7980 | 0.3759 | 0.1447 | <0.0001 | 0.5073 | 0.9245 |
| Elastic net | 0.7175 | 0.4271 | 0.1256 | <0.0001 | 0.4367 | 0.8305 |
| RF | 0.0553 | 0.4453 | 0.0142 | <0.0001 | 0.3882 | 0.7085 |
| SVM | 0.0052 | 0.0003 | 0.1747 | 0.2139 | <0.0001 | <0.0001 |
| XGBoost | 0.0256 | 0.2464 | 0.0060 | <0.0001 | 0.1341 | 0.6515 |
| **3 month** |  |  |  |  |  |  |
| Lasso | 0.1169 | 0.0069 | 0.0509 | <0.0001 | 0.0025 | 0.0018 |
| Ridge | 0.1086 | 0.0072 | 0.0522 | 0.0002 | 0.0505 | 0.0748 |
| Elastic net | 0.1118 | 0.0068 | 0.0504 | <0.0001 | 0.0026 | 0.0019 |
| RF | 0.2194 | 0.0572 | 0.1160 | 0.0110 | 0.4430 | 0.6326 |
| SVM | 0.6603 | 0.5236 | 0.8978 | 0.4204 | 0.1464 | 0.0673 |
| XGBoost | 0.0721 | 0.0140 | 0.0303 | 0.0002 | 0.0206 | 0.1102 |
| **12 month** |  |  |  |  |  |  |
| Lasso | 0.2560 | 0.0102 | 0.0010 | <0.0001 | 0.0195 | 0.0033 |
| Ridge | 0.1871 | 0.0065 | 0.0008 | <0.0001 | 0.0284 | 0.0058 |
| Elastic net | 0.2522 | 0.0134 | 0.0006 | <0.0001 | 0.0217 | 0.0039 |
| RF | 0.5023 | 0.0585 | 0.0005 | <0.0001 | 0.3821 | 0.1763 |
| SVM | 0.0332 | 0.3720 | 0.6728 | 0.9964 | 0.0026 | 0.0094 |
| XGBoost | 0.8970 | 0.0969 | 0.0054 | <0.0001 | 0.0633 | 0.0090 |

Lasso, Logistic regression with L1 penalty; Ridge, Logistic regression with L2 penalty; Elastic net, Logistic regression with Elastic net penalty; RF, Random Forest; SVM, Support Vector Machine; XGBoost, Extreme Gradient Boosting; TIMI; Global registry of acute coronary events, GRACE; Acute coronary treatment and intervention outcomes network – Get With The Guidelines, ACTION-GWTG

* Traditional models were modified using the recalculated parameters for TIMI, GRACE, and ACTION-GWTG

Supplementary Table 4. Performance of the mortality prediction models in an ST-segment elevation myocardial infarction using the traditional and optional features

|  |  | AUC (95% CI) | Specificity | Sensitivity | Accuracy | F1-score |
| --- | --- | --- | --- | --- | --- | --- |
| **In-hospital mortality** | |  |  |  |  |  |
|  | **Machine learning algorithms** | |  |  |  |  |
|  | LR with Lasso | 0.897 (0.860 – 0.933) | 0.846 | 0.774 | 0.842 | 0.353 |
|  | LR with Ridge | 0.895 (0.859 – 0.931) | 0.795 | 0.855 | 0.798 | 0.320 |
|  | LR with Elastic net | 0.897 (0.861 – 0.934) | 0.845 | 0.790 | 0.842 | **0.358** |
|  | RF | 0.901 (0.864 – 0.938) | 0.796 | 0.855 | 0.799 | 0.322 |
|  | SVM | 0.847 (0.781 – 0.914) | 0.820 | 0.79 | 0.819 | 0.327 |
|  | XGBoost | **0.911 (0.883 – 0.940)** | 0.848 | 0.839 | 0.847 | 0.379 |
|  | **Traditional and modified traditional model** | |  |  |  |  |
|  | TIMI | 0.855 (0.813 – 0.897) | 0.769 | 0.774 | 0.769 | 0.272 |
|  | GRACE | **0.896 (0.862 – 0.930)** | 0.842 | 0.774 | 0.838 | **0.347** |
|  | ACTION-GWTG | 0.891 (0.855 – 0.927) | 0.837 | 0.758 | 0.832 | 0.335 |
|  | Modified TIMI* | 0.885 (0.849 – 0.920) | 0.826 | 0.806 | 0.825 | 0.339 |
|  | Modified GRACE* | 0.901 (0.870 – 0.932) | 0.826 | 0.823 | 0.826 | 0.345 |
|  | Modified ACTION-GWTG* | 0.859 (0.810 – 0.907) | 0.833 | 0.710 | 0.826 | 0.312 |
| **3 month mortality** | |  |  |  |  |  |
|  | **Machine learning algorithms** | |  |  |  |  |
|  | LR with Lasso | 0.788 (0.687 – 0.889) | 0.666 | 0.857 | 0.670 | 0.099 |
|  | LR with Ridge | 0.780 (0.667 – 0.894) | 0.738 | 0.714 | 0.737 | 0.103 |
|  | LR with Elastic net | 0.793 (0.697 – 0.889) | 0.676 | 0.857 | 0.680 | 0.102 |
|  | RF | **0.813 (0.725 – 0.901)** | 0.819 | 0.667 | 0.816 | **0.133** |
|  | SVM | 0.730 (0.635 – 0.824) | 0.638 | 0.714 | 0.639 | 0.077 |
|  | XGBoost | 0.794 (0.699 – 0.890) | 0.622 | 0.905 | 0.628 | 0.093 |
|  | **Traditional and modified traditional model** | |  |  |  |  |
|  | TIMI | 0.743 (0.650 – 0.837) | 0.610 | 0.810 | 0.614 | 0.082 |
|  | GRACE | **0.766 (0.670 – 0.862)** | 0.652 | 0.857 | 0.657 | **0.096** |
|  | ACTION-GWTG | 0.709 (0.602 – 0.816) | 0.630 | 0.667 | 0.63 | 0.070 |
|  | Modified TIMI* | 0.704 (0.593 – 0.815) | 0.628 | 0.714 | 0.629 | 0.075 |
|  | Modified GRACE* | 0.602 (0.458 - 0.745) | 0.832 | 0.238 | 0.820 | 0.053 |
|  | Modified ACTION-GWTG* | 0.653 (0.528 – 0.778) | 0.731 | 0.476 | 0.726 | 0.068 |
| **12 month mortality** | |  |  |  |  |  |
|  | **Machine learning algorithms** | |  |  |  |  |
|  | LR with Lasso | **0.835 (0.774 – 0.897)** | 0.806 | 0.719 | 0.803 | 0.191 |
|  | LR with Ridge | 0.828 (0.769 – 0.887) | 0.798 | 0.750 | 0.797 | 0.193 |
|  | LR with Elastic net | 0.830 (0.767 – 0.892) | 0.866 | 0.562 | 0.856 | **0.202** |
|  | RF | 0.824 (0.764 – 0.884) | 0.833 | 0.562 | 0.824 | 0.171 |
|  | SVM | 0.710 (0.625 – 0.795) | 0.554 | 0.750 | 0.560 | 0.099 |
|  | XGBoost | 0.816 (0.749 – 0.884) | 0.833 | 0.531 | 0.823 | 0.163 |
|  | **Traditional and modified traditional model** | |  |  |  |  |
|  | TIMI | 0.793 (0.726 – 0.860) | 0.642 | 0.844 | 0.648 | 0.134 |
|  | GRACE | **0.826 (0.770 – 0.881)** | 0.677 | 0.812 | 0.681 | **0.142** |
|  | ACTION-GWTG | 0.780 (0.709 – 0.850) | 0.770 | 0.562 | 0.763 | 0.134 |
|  | Modified TIMI* | 0.802 (0.736 – 0.868) | 0.786 | 0.688 | 0.783 | 0.170 |
|  | Modified GRACE* | 0.741 (0.663 – 0.820) | 0.771 | 0.625 | 0.766 | 0.148 |
|  | Modified ACTION-GWTG* | 0.659 (0.554 – 0.764) | 0.748 | 0.531 | 0.741 | 0.117 |

AUC, area under the receiver operating characteristic curve; LR, Logistic regression; Lasso, L1 penalty; Ridge, L2 penalty; Elastic net, Elastic net penalty; RF, Random Forest; SVM, Support Vector Machine; XGBoost, Extreme Gradient Boosting

* Traditional models were modified using the recalculated parameters for TIMI, GRACE, and ACTION-GWTG

Supplementary Table 5. Performance of the mortality prediction models in a Non-ST-segment elevation myocardial infarction using the traditional and optional features

|  |  | AUC (95% CI) | Specificity | Sensitivity | Accuracy | F1-score |
| --- | --- | --- | --- | --- | --- | --- |
| **In-hospital mortality** | |  |  |  |  |  |
|  | **Machine learning algorithms** | |  |  |  |  |
|  | LR with Lasso | **0.887 (0.854 – 0.920)** | 0.771 | 0.879 | 0.775 | **0.232** |
|  | LR with Ridge | 0.885 (0.850 – 0.920) | 0.722 | 0.909 | 0.729 | 0.207 |
|  | LR with Elastic net | **0.887 (0.854 – 0.920)** | 0.742 | 0.879 | 0.747 | 0.212 |
|  | RF | 0.878 (0.842 – 0.914) | 0.749 | 0.803 | 0.751 | 0.200 |
|  | SVM | 0.770 (0.709 – 0.831) | 0.716 | 0.682 | 0.715 | 0.157 |
|  | XGBoost | 0.879 (0.846 – 0.913) | 0.710 | 0.864 | 0.716 | 0.191 |
|  | **Traditional and modified traditional model** | |  |  |  |  |
|  | TIMI | 0.669 (0.613 – 0.724) | 0.686 | 0.576 | 0.682 | 0.123 |
|  | GRACE | **0.873 (0.840 – 0.906)** | 0.734 | 0.803 | 0.736 | 0.191 |
|  | ACTION-GWTG | 0.871 (0.836 – 0.907) | 0.812 | 0.712 | 0.808 | **0.224** |
|  | Modified TIMI* | 0.709 (0.656 – 0.763) | 0.506 | 0.788 | 0.516 | 0.112 |
|  | Modified GRACE* | 0.876 (0.841 – 0.912) | 0.806 | 0.773 | 0.805 | 0.235 |
|  | Modified ACTION-GWTG* | 0.884 (0.851 – 0.916) | 0.819 | 0.758 | 0.817 | 0.243 |
| **3 month mortality** | |  |  |  |  |  |
|  | **Machine learning algorithms** | |  |  |  |  |
|  | LR with Lasso | 0.852 (0.801 – 0.904) | 0.709 | 0.861 | 0.712 | 0.124 |
|  | LR with Ridge | 0.846 (0.790 – 0.903) | 0.749 | 0.806 | 0.750 | **0.132** |
|  | LR with Elastic net | **0.855 (0.804 – 0.906)** | 0.764 | 0.750 | 0.763 | 0.130 |
|  | RF | 0.823 (0.752 – 0.894) | 0.643 | 0.833 | 0.647 | 0.100 |
|  | SVM | 0.733 (0.652 – 0.813) | 0.670 | 0.639 | 0.669 | 0.083 |
|  | XGBoost | 0.834 (0.774 – 0.894) | 0.654 | 0.861 | 0.659 | 0.106 |
|  | **Traditional and modified traditional model** | |  |  |  |  |
|  | TIMI | 0.672 (0.592 – 0.751) | 0.689 | 0.528 | 0.685 | 0.073 |
|  | GRACE | 0.777 (0.711 – 0.844) | 0.705 | 0.694 | 0.704 | 0.100 |
|  | ACTION-GWTG | **0.795 (0.728 – 0.862)** | 0.726 | 0.750 | 0.727 | **0.114** |
|  | Modified TIMI* | 0.675 (0.596 – 0.754) | 0.534 | 0.750 | 0.539 | 0.071 |
|  | Modified GRACE* | 0.774 (0.709 – 0.838) | 0.623 | 0.778 | 0.627 | 0.089 |
|  | Modified ACTION-GWTG* | 0.782 (0.721 – 0.843) | 0.759 | 0.639 | 0.756 | 0.11 |
| **12 month mortality** | |  |  |  |  |  |
|  | **Machine learning algorithms** | |  |  |  |  |
|  | LR with Lasso | 0.864 (0.835 – 0.894) | 0.712 | 0.887 | 0.720 | 0.227 |
|  | LR with Ridge | 0.863 (0.832 – 0.893) | 0.748 | 0.803 | 0.750 | **0.229** |
|  | LR with Elastic net | **0.865 (0.835 – 0.894)** | 0.718 | 0.873 | 0.725 | 0.227 |
|  | RF | 0.846 (0.810 – 0.882) | 0.708 | 0.873 | 0.716 | 0.221 |
|  | SVM | 0.747 (0.690 – 0.803) | 0.757 | 0.620 | 0.750 | 0.186 |
|  | XGBoost | 0.861 (0.828 – 0.894) | 0.752 | 0.845 | 0.756 | 0.242 |
|  | **Traditional and modified traditional model** | |  |  |  |  |
|  | TIMI | 0.675 (0.619 – 0.731) | 0.695 | 0.549 | 0.688 | 0.140 |
|  | GRACE | **0.808 (0.764 – 0.852)** | 0.697 | 0.789 | 0.701 | **0.196** |
|  | ACTION-GWTG | 0.790 (0.740 – 0.839) | 0.719 | 0.718 | 0.719 | 0.191 |
|  | Modified TIMI* | 0.729 (0.683 – 0.776) | 0.545 | 0.845 | 0.559 | 0.150 |
|  | Modified GRACE* | 0.820 (0.779 – 0.861) | 0.715 | 0.761 | 0.717 | 0.199 |
|  | Modified ACTION-GWTG* | 0.808 (0.768 – 0.848) | 0.739 | 0.732 | 0.739 | 0.206 |

AUC, area under the receiver operating characteristic curve; LR, Logistic regression; Lasso, L1 penalty; Ridge, L2 penalty; Elastic net, Elastic net penalty; RF, Random Forest; SVM, Support Vector Machine; XGBoost, Extreme Gradient Boosting

* Traditional models were modified using the recalculated parameters for TIMI, GRACE, and ACTION-GWTG

Supplementary Table 6. Performance of the mortality prediction models in an acute myocardial infarction using the traditional, optional variables and medications at discharge

|  | AUC | | Accuracy | | F1-score | |
| --- | --- | --- | --- | --- | --- | --- |
|  | Without medication | With medication | Without medication | With medication | Without medication | With medication |
| **STEMI** |  |  |  |  |  |  |
| **3 months mortality** |  |  |  |  |  |  |
| LR with Lasso | 0.788 | 0.788 | 0.670 | 0.663 | 0.099 | 0.097 |
| LR with Ridge | 0.780 | 0.807 | 0.737 | 0.878 | 0.103 | 0.165 |
| LR with Elastic net | 0.793 | 0.788 | 0.680 | 0.666 | 0.102 | 0.093 |
| RF | 0.813 | 0.780 | 0.816 | 0.797 | 0.133 | 0.106 |
| SVM | 0.730 | 0.665 | 0.639 | 0.731 | 0.077 | 0.082 |
| XGboost | 0.794 | 0.813 | 0.628 | 0.656 | 0.093 | 0.100 |
| **12 months mortality** |  |  |  |  |  |  |
| LR with Lasso | 0.835 | 0.783 | 0.803 | 0.771 | 0.191 | 0.144 |
| LR with Ridge | 0.828 | 0.787 | 0.797 | 0.721 | 0.193 | 0.143 |
| LR with Elastic net | 0.830 | 0.784 | 0.856 | 0.800 | 0.202 | 0.147 |
| RF | 0.824 | 0.795 | 0.824 | 0.794 | 0.171 | 0.164 |
| SVM | 0.710 | 0.717 | 0.560 | 0.813 | 0.099 | 0.089 |
| XGboost | 0.816 | 0.804 | 0.823 | 0.818 | 0.163 | 0.159 |
| **NSTEMI** |  |  |  |  |  |  |
| **3 months mortality** |  |  |  |  |  |  |
| LR with Lasso | 0.852 | 0.864 | 0.712 | 0.714 | 0.124 | 0.128 |
| LR with Ridge | 0.846 | 0.856 | 0.750 | 0.741 | 0.132 | 0.132 |
| LR with Elastic net | 0.855 | 0.865 | 0.763 | 0.719 | 0.130 | 0.130 |
| RF | 0.823 | 0.846 | 0.647 | 0.721 | 0.100 | 0.120 |
| SVM | 0.733 | 0.767 | 0.669 | 0.700 | 0.083 | 0.102 |
| XGboost | 0.834 | 0.834 | 0.659 | 0.686 | 0.106 | 0.118 |
| **12 months mortality** |  |  |  |  |  |  |
| LR with Lasso | 0.864 | 0.863 | 0.720 | 0.789 | 0.227 | 0.239 |
| LR with Ridge | 0.863 | 0.856 | 0.750 | 0.745 | 0.229 | 0.222 |
| LR with Elastic net | 0.865 | 0.862 | 0.725 | 0.765 | 0.227 | 0.236 |
| RF | 0.846 | 0.848 | 0.716 | 0.684 | 0.221 | 0.211 |
| SVM | 0.747 | 0.782 | 0.750 | 0.664 | 0.186 | 0.176 |
| XGboost | 0.861 | 0.865 | 0.756 | 0.718 | 0.242 | 0.225 |

AUC, area under the receiver operating characteristic curve; LR, Logistic regression; Lasso, L1 penalty; Ridge, L2 penalty; Elastic net, Elastic net penalty; RF, Random Forest; SVM, Support Vector Machine; XGBoost, Extreme Gradient Boosting

‘Without medication’ means the machine learning-based models including the traditional and optional variables, whereas ‘With medication’ denotes those including the traditional, optional, and medication variables.

Supplementary Table 7. Importance of the predictors in the best performing models, including traditional and optional variables in an ST-segment elevation myocardial infarction

| Type | Variables | In-hospital mortality | 3-month mortality | 12-month mortality |
| --- | --- | --- | --- | --- |
|  |  | XGBoost | RF | Lasso |
| Traditional | Sex | O (0.001) | 0.98 (19) | X |
|  | Age | O (0.104) | 6.33 (2) | O (0.053) |
|  | Door-to-Balloon time | O (0.014) | 4.29 (11) | X |
|  | Weight | O (0.007) | 4.97 (8) | X |
|  | Cardiac arrest before ED arrival | O (0.046) | 0.95 (21) | X |
|  | Hypertension | O (0.001) | 1.09 (16) | X |
|  | Diabetes mellitus | X | 0.96 (20) | X |
|  | Previous MI | X | 0.37 (39) | X |
|  | Previous PCI | X | 0.45 (38) | X |
|  | Systolic BP | O (0.077) | 4.74 (9) | X |
|  | Diastolic BP | O (0.016) | 4.52 (10) | X |
|  | Heart rate | O (0.057) | 6.29 (3) | O (0.007) |
|  | Cardiogenic shock | O (0.260) | 0.51 (35) | X |
|  | Heart failure | O (0.047) | 0.83 (24) | X |
|  | Creatinine | O (0.162) | 5.72 (7) | X |
|  | Hemoglobin | O (0.114) | 5.76 (6) | O (-0.169) |
|  | Troponin | O (0.007) | 0.76 (27) | X |
|  | Three vessel disease | Not applicable | 0.28 (40) | X |
|  | LV ejection fraction | Not applicable | 6.36 (1) | O (-0.006) |
| Optional | Height | O (0.012) | 4.25 (12) | X |
|  | Chest pain | O (0.045) | 0.81 (25) | O (-0.410) |
|  | Dyspnea | O (0.007) | 1.08 (17) | X |
|  | Loss of awareness | O (0.011) | 0.61 (32) | X |
|  | Sweat | X | 1.00 (18) | O (-0.016) |
|  | Vertigo and systemic weakness | O (0.001) | 0.74 (28) | X |
|  | Epigastric pain | O (0.001) | 0.48 (37) | X |
|  | Radiating pain | X | 0.73 (29) | X |
|  | Smoking | O (0.007) | 2.14 (13) | O (0.422) |
|  | Dyslipidemia | X | 0.49 (36) | X |
|  | Previous stroke | X | 0.65 (31) | X |
|  | Atrial fibrillation at arrival | O (0.003) | 0.25 (41) | X |
|  | Culprit lesion | Not applicable | 1.56 (14) | X |
|  | Stenosis of the left main artery | Not applicable | 0.19 (42) | X |
|  | Stenosis of pLAD | Not applicable | 1.10 (15) | O (0.058) |
|  | Complete revascularization | Not applicable | 0.77 (26) | X |
|  | Stent insertion | Not applicable | 0.59 (33) | X |
|  | Cardiac arrest during admission | Not applicable | 0.17 (43) | X |
|  | Cardiogenic shock during admission | Not applicable | 0.68 (30) | X |
|  | Atrial fibrillation during admission | Not applicable | 0.84 (22) | O (0.463) |
|  | Heart failure during admission | Not applicable | 0.84 (22) | X |
|  | Peak creatinine | Not applicable | 6.02 (5) | O (0.115) |
|  | Bottom hemoglobin | Not applicable | 6.22 (4) | O (-0.148) |
|  | Atrial fibrillation at discharge | Not applicable | 0.58 (34) | X |
|  | Peak troponin | Not applicable | 0.01 (44) | X |

Variable importance denotes Gain in XGBoost, Mean Decrease Gini, and rank in parentheses in RF and the value of coefficients in Lasso.

For in-hospital mortality, the variables during admission and at discharge are not included in the predictive model and are indicated in the table as ‘Not applicable’.

Supplementary Table 8. Importance of predictors in the best performing models including traditional and optional variables in a Non-ST-segment elevation myocardial infarction

| Type | Variables | In-hospital mortality | 3-month mortality | 12-month mortality |
| --- | --- | --- | --- | --- |
|  |  | Lasso | Elastic net | Elastic net |
| Core | Male | X | X | X |
|  | Age | O (0.048) | O (0.025) | O (0.034) |
|  | Weight | X | O (0.086) | O (-0.017) |
|  | Cardiac arrest before ED arrival | O (0.980) | X | O (-0.496) |
|  | Hypertension | O (0.082) | X | X |
|  | Diabetes mellitus | O (0.152) | O (0.192) | O (0.127) |
|  | Previous MI | X | X | O (0.580) |
|  | Previous PCI | X | X | X |
|  | Systolic BP | O (-0.014) | X | O (-0.002) |
|  | Diastolic BP | O (-0.001) | O (-0.008) | O (-0.010) |
|  | Heart rate | O (0.007) | O (0.008) | O (0.010) |
|  | ECG | O (0.014) | O (-0.033) | O (-0.437) |
|  | Cardiogenic shock | O (1.411) | O (-0.080) | O (-0.447) |
|  | Heart failure | O (0.505) | O (0.022) | O (0.078) |
|  | Creatinine | O (0.050) | X | X |
|  | Hemoglobin | O (-0.088) | O (-0.041) | O (-0.067) |
|  | Troponin | X | X | O (0.106) |
|  | Three vessel disease | Not applicable | O (0.035) | O (0.423) |
|  | LV ejection fraction | Not applicable | O (-0.033) | O (-0.015) |
| Optional | Chest pain | O (-0.162) | X | O (-0.225) |
|  | Dyspnea | O (0.272) | O (0.086) | O (0.329) |
|  | Loss of awareness | X | X | X |
|  | Sweat | O (-0.152) | X | X |
|  | Vertigo and systemic weakness | X | O (0.245) | X |
|  | Epigastric pain | O (0.605) | X | X |
|  | Radiating pain | O (-0.365) | X | X |
|  | Height | X | X | O (0.015) |
|  | Smoking | O (-0.106) | X | X |
|  | Dyslipidemia | X | O (-0.135) | O (-0.121) |
|  | Previous stroke | O (0.179) | X | O (0.357) |
|  | Atrial fibrillation at arrival | O (-0.132) | X | O (0.011) |
|  | Cardiac arrest during admission | Not applicable | X | X |
|  | Cardiogenic shock during admission | Not applicable | O (0.082) | O (0.112) |
|  | Atrial fibrillation during admission | Not applicable | O (0.272) | O (0.176) |
|  | Heart failure during admission | Not applicable | O (0.164) | O (0.155) |
|  | Peak creatinine | Not applicable | O (0.092) | O (0.103) |
|  | Bottom hemoglobin | Not applicable | O (-0.101) | O (-0.124) |
|  | Atrial fibrillation at discharge | Not applicable | X | O (0.078) |
|  | Peak troponin | Not applicable | X | X |

Variable importance denotes the value of coefficients in Lasso and elastic net.

For in-hospital mortality, variables during admission and at discharge are not included in the predictive model and are indicated in the table as ‘Not applicable’.

Supplementary Table 9. Performance of the mortality prediction models in ST-segment elevation myocardial infarction considering data imbalance

| STEMI | Predictors | Model | AUC | | | |
| --- | --- | --- | --- | --- | --- | --- |
|  |  |  | Original | Up-sampling | Down-sampling | SMOTE |
| In-hospital mortality | N | - | 4443 | 8464 | 422 | 1477 |
|  | Traditional | LR with Lasso | 0.890 | 0.900 | 0.903 | 0.894 |
|  |  | LR with Ridge | 0.889 | 0.893 | 0.899 | 0.893 |
|  |  | LR with Elastic net | 0.890 | 0.899 | 0.895 | 0.894 |
|  |  | RF | 0.910 | 0.910 | 0.904 | **0.917** |
|  |  | SVM | 0.819 | 0.904 | 0.897 | 0.901 |
|  |  | **XGBoost** | **0.912** | 0.905 | 0.905 | 0.910 |
|  | Traditional + optional | LR with Lasso | 0.897 | 0.900 | 0.901 | 0.897 |
|  |  | LR with Ridge | 0.895 | 0.898 | 0.893 | 0.894 |
|  |  | LR with Elastic net | 0.897 | 0.899 | 0.900 | 0.898 |
|  |  | RF | 0.901 | 0.911 | 0.903 | 0.906 |
|  |  | SVM | 0.847 | 0.904 | 0.900 | 0.897 |
|  |  | **XGBoost** | **0.911** | 0.895 | 0.902 | 0.909 |
| 3 month mortality | N | - | 3918 | 7742 | 94 | 329 |
|  | Traditional | LR with Lasso | 0.777 | 0.776 | 0.777 | 0.706 |
|  |  | LR with Ridge | 0.779 | 0.777 | 0.678 | 0.721 |
|  |  | LR with Elastic net | 0.777 | 0.778 | 0.715 | 0.732 |
|  |  | RF | 0.763 | 0.750 | 0.784 | **0.786** |
|  |  | SVM | 0.667 | 0.760 | 0.763 | 0.766 |
|  |  | **XGBoost** | **0.784** | 0.657 | 0.778 | 0.781 |
|  | Traditional + optional | LR with Lasso | 0.788 | 0.779 | 0.768 | 0.734 |
|  |  | LR with Ridge | 0.780 | 0.789 | 0.788 | 0.759 |
|  |  | LR with Elastic net | 0.793 | 0.786 | 0.773 | 0.757 |
|  |  | **RF** | **0.813** | 0.758 | 0.794 | 0.805 |
|  |  | SVM | 0.730 | 0.771 | 0.688 | 0.776 |
|  |  | XGBoost | 0.794 | 0.695 | 0.766 | 0.750 |
| 12 month mortality | N | - | 3922 | 7668 | 176 | 616 |
|  | Traditional | LR with Lasso | 0.835 | 0.842 | 0.824 | 0.824 |
|  |  | **LR with Ridge** | **0.840** | 0.839 | 0.834 | 0.817 |
|  |  | LR with Elastic net | 0.835 | 0.842 | 0.828 | 0.822 |
|  |  | RF | 0.825 | 0.829 | 0.830 | 0.832 |
|  |  | SVM | 0.684 | **0.844** | 0.838 | 0.811 |
|  |  | XGBoost | 0.806 | 0.759 | 0.826 | 0.831 |
|  | Traditional + optional | **LR with Lasso** | **0.835** | **0.847** | 0.817 | 0.826 |
|  |  | LR with Ridge | 0.828 | 0.834 | 0.814 | 0.806 |
|  |  | LR with Elastic net | 0.830 | 0.847 | 0.807 | 0.828 |
|  |  | RF | 0.824 | 0.819 | 0.823 | 0.817 |
|  |  | SVM | 0.710 | 0.839 | 0.821 | 0.822 |
|  |  | XGBoost | 0.816 | 0.780 | 0.806 | 0.830 |

AUC, area under the receiver operating characteristic curve; LR, Logistic regression; Lasso, L1 penalty; Ridge, L2 penalty; Elastic net, Elastic net penalty; RF, Random Forest; SVM, Support Vector Machine; XGBoost, Extreme Gradient Boosting; SMOTE, Synthetic minority oversampling technique

Supplementary Table 10. Performance of the mortality prediction models in a Non-ST-segment elevation myocardial infarction considering data imbalance

| STEMI | Predictors | Model | AUC | | | |
| --- | --- | --- | --- | --- | --- | --- |
|  |  |  | Original | Up-sampling | Down-sampling | SMOTE |
| In-hospital mortality | N | - | 6926 | 13422 | 430 | 1505 |
|  | Traditional | LR with Lasso | 0.886 | 0.882 | 0.884 | 0.884 |
|  |  | LR with Ridge | 0.885 | 0.882 | 0.883 | 0.882 |
|  |  | LR with Elastic net | 0.886 | 0.883 | 0.884 | 0.884 |
|  |  | **RF** | **0.889** | 0.875 | **0.894** | 0.885 |
|  |  | SVM | 0.760 | 0.878 | 0.882 | 0.877 |
|  |  | XGBoost | 0.888 | 0.871 | 0.877 | 0.884 |
|  | Traditional + optional | **LR with Lasso** | **0.887** | 0.879 | 0.867 | 0.860 |
|  |  | LR with Ridge | 0.885 | 0.878 | 0.870 | 0.862 |
|  |  | LR with Elastic net | 0.887 | 0.879 | 0.866 | 0.863 |
|  |  | RF | 0.878 | 0.869 | **0.888** | 0.872 |
|  |  | SVM | 0.770 | 0.871 | 0.871 | 0.870 |
|  |  | XGBoost | 0.879 | 0.857 | 0.873 | 0.866 |
| 3 month mortality | N | - | 6187 | 12162 | 212 | 742 |
|  | Traditional | **LR with Lasso** | **0.849** | 0.845 | 0.805 | 0.818 |
|  |  | LR with Ridge | 0.826 | 0.841 | 0.822 | 0.826 |
|  |  | LR with Elastic net | 0.849 | 0.845 | 0.810 | 0.820 |
|  |  | RF | 0.799 | 0.818 | 0.819 | 0.807 |
|  |  | SVM | 0.715 | 0.843 | 0.832 | 0.825 |
|  |  | XGBoost | 0.824 | 0.787 | 0.796 | 0.793 |
|  | Traditional + optional | LR with Lasso | 0.852 | 0.860 | 0.825 | 0.845 |
|  |  | **LR with Ridge** | 0.846 | 0.856 | 0.842 | 0.845 |
|  |  | LR with Elastic net | **0.855** | 0.860 | 0.832 | 0.846 |
|  |  | RF | 0.823 | 0.813 | 0.821 | 0.817 |
|  |  | SVM | 0.733 | **0.863** | 0.775 | 0.836 |
|  |  | XGBoost | 0.834 | 0.798 | 0.812 | 0.805 |
| 12 month mortality | N | - | 6178 | 11886 | 470 | 1645 |
|  | Traditional | **LR with Lasso** | **0.860** | 0.860 | 0.861 | 0.849 |
|  |  | LR with Ridge | 0.858 | 0.860 | 0.847 | 0.854 |
|  |  | LR with Elastic net | 0.859 | 0.860 | 0.860 | 0.842 |
|  |  | RF | 0.836 | 0.854 | 0.859 | 0.860 |
|  |  | SVM | 0.729 | **0.862** | 0.858 | 0.857 |
|  |  | XGBoost | 0.851 | 0.856 | 0.856 | 0.861 |
|  | Traditional + optional | LR with Lasso | 0.864 | 0.861 | 0.862 | 0.851 |
|  |  | LR with Ridge | 0.863 | 0.859 | 0.856 | 0.855 |
|  |  | **LR with Elastic net** | **0.865** | 0.862 | **0.870** | 0.852 |
|  |  | RF | 0.846 | 0.855 | 0.865 | 0.861 |
|  |  | SVM | 0.747 | 0.865 | 0.864 | 0.863 |
|  |  | XGBoost | 0.861 | 0.858 | 0.854 | 0.863 |

AUC, area under the receiver operating characteristic curve; LR, Logistic regression; Lasso, L1 penalty; Ridge, L2 penalty; Elastic net, Elastic net penalty; RF, Random Forest; SVM, Support Vector Machine; XGBoost, Extreme Gradient Boosting; SMOTE, Synthetic minority oversampling technique

Supplementary Table 11. Matrix of the traditional and optional features for predicting the in-hospital, three- month and 12-month mortality

|  |  | Traditional variables | Optional variables |
| --- | --- | --- | --- |
| Pre-ED | Demographic characteristics | Sex, age, weight | Height |
|  | Initial symptoms |  | Chest pain, shortness of breath, LOC, sweating, general weakness, epigastric pain, radiating pain |
|  | History | Hypertension, diabetes mellitus, prior MI, prior PCI, prior PAD | Smoking, dyslipidemia, prior stroke |
|  | Pre-ED event | Cardiac arrest |  |
| ED | Clinical presentation | SBP, DBP, HR, cardiogenic shock, CHF |  |
|  | Electrocardiographic findings |  | ECG, AF |
|  | Initial laboratory findings |  | Troponin, creatinine, hemoglobin |
| Admission | Angiographic findings | Three vessel disease | Culprit lesion, stenosis of the left main artery, stenosis of pLAD, complete revascularization, stent |
|  | Events during hospital admission |  | Cardiac arrest, cardiogenic shock, AF, CHF |
|  | Peak laboratory findings |  | Peak Troponin, peak creatinine, bottom hemoglobin |
|  | Echocardiographic findings | EF |  |
|  | Electrocardiographic findings |  | AF |
| Discharge^*^ | Medication |  | Aspirin, clopidogrel, prasugrel, ticagrelor, CCB, BB, ACEi, ARB, statin, ezetimide, warfarin, NOAC, OHA |

AF, atrial fibrillation; ACEi, angiotensin-converting enzyme inhibitor; ARB, angiotensin receptor blocker; BB, beta-blocker; CCB, calcium channel blocker; CHF, congestive heart failure; DBP, diastolic blood pressure; ECG, electrocardiogram; ED, emergency department; EF, ejection fraction; HR, heart rate; LOC, loss of consciousness; MI, myocardial infarction; NOAC, non-vitamin K antagonist oral anticoagulants; OHA, oral hypoglycemic agent; PAD, peripheral artery disease; PCI, percutaneous coronary intervention; pLAD, proximal left descending artery; SBP, systolic blood pressure

^*^ Medications at discharge were considered additionally in the model, including the traditional and optional variables.
